# Functional Gene Network of Prenyltransferases in *Arabidopsis thaliana*

**DOI:** 10.3390/molecules24244556

**Published:** 2019-12-12

**Authors:** Diana Kopcsayová, Eva Vranová

**Affiliations:** 1 Institute of Biology and Ecology, Pavol Jozef Šafárik University in Košice, 04180 Košice, Slovakia; 2 Institute of Pharmacology, University of Veterinary Medicine and Pharmacy in Košice, 04181 Košice, Slovakia

**Keywords:** prenyltransferases, isoprenoid diphosphate synthases, product specificity, co-expression analysis, mutants, *Arabidopsis*

## Abstract

Prenyltransferases (PTs) are enzymes that catalyze prenyl chain elongation. Some are highly similar to each other at the amino acid level. Therefore, it is difficult to assign their function based solely on their sequence homology to functional orthologs. Other experiments, such as in vitro enzymatic assay, mutant analysis, and mutant complementation are necessary to assign their precise function. Moreover, subcellular localization can also influence the functionality of the enzymes within the pathway network, because different isoprenoid end products are synthesized in the cytosol, mitochondria, or plastids from prenyl diphosphate (prenyl-PP) substrates. In addition to in vivo functional experiments, in silico approaches, such as co-expression analysis, can provide information about the topology of PTs within the isoprenoid pathway network. There has been huge progress in the last few years in the characterization of individual *Arabidopsis* PTs, resulting in better understanding of their function and their topology within the isoprenoid pathway. Here, we summarize these findings and present the updated topological model of PTs in the *Arabidopsis thaliana* isoprenoid pathway.

## 1. Introduction

Isoprenoids form a large group of more than 55,000 natural metabolites despite the fact that they are all derived from just five carbon units of isopentenyl diphosphate (IPP) [1]. IPP and its isomer dimethylallyl diphosphate (DMAPP) are synthetized in a highly conserved manner in almost all living species. In plants, they are produced in the cytosol via the mevalonic acid (MVA) pathway and in plastids through the MVA-independent methylerythritol phosphate (MEP) pathway. However, the vast majority of organisms possess just one of these pathways—the MVA pathway is more widely spread, utilized by archaebacteria, some Gram-positive bacteria, yeasts, fungi, and animals, and the MEP pathway provides the isoprenoid precursors in bacteria, cyanobacteria, and green algae [2].

**Figure 1:**
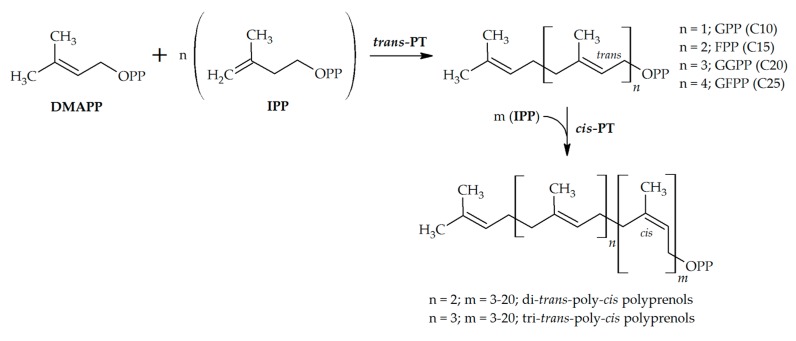
Schematic representation of the reactions catalyzed by *trans*-prenyltransferases and *cis*-prenyltransferases showing the stereochemistry of the double bond formed in the enzyme product. DMAPP, dimethylallyl diphosphate; IPP, isopentenyl diphosphate; PT, prenyltransferase; GPP, geranyl diphosphate; FPP, farnesyl diphosphate; GGPP, geranylgeranyl diphosphate; GFPP, geranylfarnesyl diphosphate; and OPP, diphosphate ester.

Some groups of isoprenoids, such as cytokinins and hemiterpenoids, are derived directly from the DMAPP, but the great structural and functional diversity of isoprenoids is created by the activity of prenyltransferases (PTs). In a broader sense, PTs are any enzymes that catalyze the transfer of the prenyl group [3]. Possible acceptors are both aliphatic and aromatic compounds with low or even high molecular weight including proteins. In a narrower sense, PTs catalyze condensation between DMAPP or another prenyl-PP, which serves as an allylic donor, and IPP, which serves as an acceptor [4] (Figure 1). These are also known in the literature as isoprenyl-PP synthases (IDSs) [5]. In this review, we will use the term PT for convenience, as PT is more frequently used in the literature, but we will refer to only the prenyl chain elongating enzymes that catalyze sequential condensation of allylic diphosphate and IPP.

**Figure 2:**
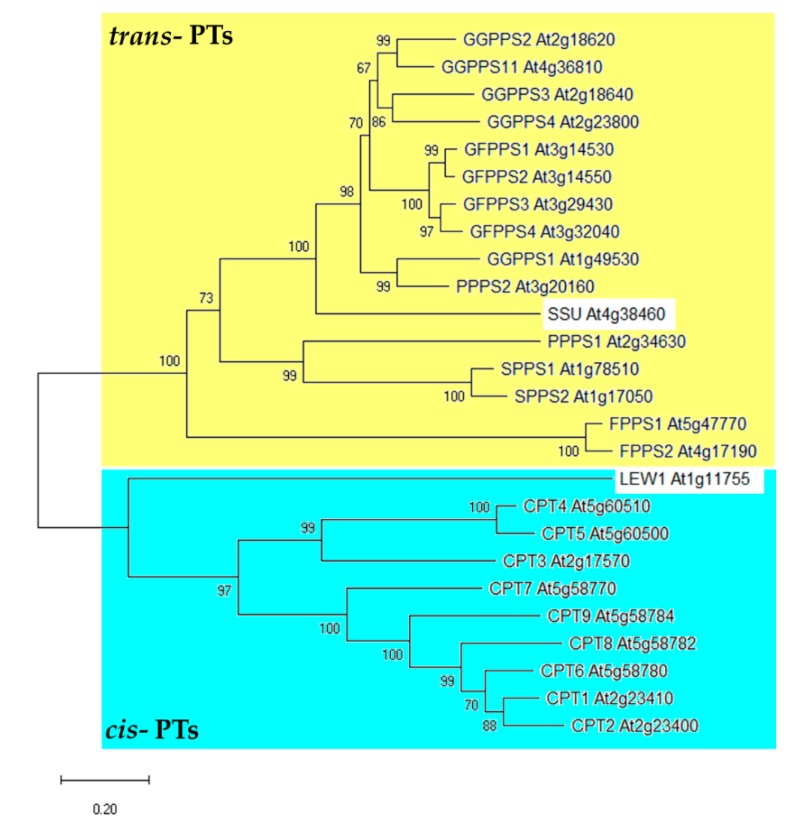
Phylogenetic analysis of the prenyltransferase family in *A. thaliana*. The phylogenetic tree illustrates the relatedness of PTs, which cluster into two major clades—*trans*- and *cis*-PTs. Enzyme abbreviations are listed in Table 1. Enzymes in white boxes are not PTs, but they interact with PTs—small subunit (SSU) with geranylgeranyl diphosphate synthase 11 (GGPPS11) [8,9] and LEW1 with *cis-*PT3 (CPT3) [10]. Because the tree was left unrooted, the branching order may not always reflect the evolutionary course that the enzymes have taken. The evolutionary analysis was conducted in MEGA X [11] using the neighbor-joining method with 500 bootstrap replicates. The scale bar represents 0.2 amino acid substitutions per site.

The binding of both co-substrates to the enzyme in the presence of the divalent Mg salts creates carbocation on the allylic substrate, followed by the electrophilic attack on the double bond of the IPP. As a result, a new carbon bond joining the co-substrates is formed [6]. The product is then released from the active site or can act as a primer in the subsequent coupling with another IPP molecule. With every further elongation step, a linear prenyl-PP grows by C_5_. The carbon chain length of the final isoprenoid polymer and its stereochemical properties are determined by the specificity of the PT, which is given to the PT by its structural features. The two main classes of PTs are recognized due to the differences in the spatial arrangement of units in the isoprenoid product, namely *trans-* or *(E)*-PTs and *cis-* or *(Z)*-PTs (Figure 1). Basically, shorter prenyl-PPs are formed via *trans*-condensation and polyprenyl-PPs via *cis*-condensation in plants [7]. Although *trans-* and *cis-*PTs catalyze similar reactions, they are evolutionarily distinct [7] (Figure 2).

## 2. Prenyltransferases in *Arabidopsis thaliana*

**Figure 3:**
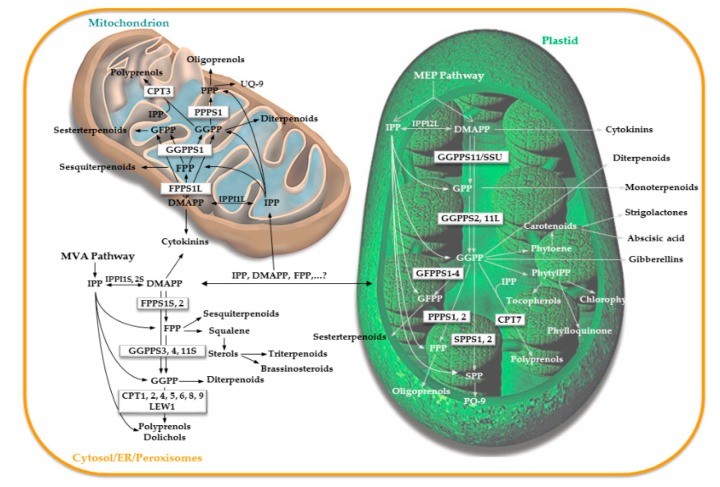
Topology of the prenyltransferase genes within the isoprenoid pathway of *A. thaliana*. Based on the isoprenoid pathway network constructed by Vranová et al. [16]. Abbreviations of the enzymes in boxes are given in Table 1. MVA, mevalonate; MEP, 2-C-methyl-D-erythritol 4-phosphate; IPPI, isopentenyl diphosphate (IPP, C5) isomerase; IPPI1S, 2S, short IPPI variants; IPPI1L, 2L, long IPPI variants; DMAPP (C5), dimethylallyl diphosphate; GPP (C10), geranyl diphosphate; FPP (C15), farnesyl diphosphate; GGPP (C20), geranylgeranyl diphosphate; GFPP (C25), geranylfarnesyl diphosphate; PPP (C ≥ 30), polyprenyl diphosphate; SPP (C45), solanesyl diphosphate; PQ-9, plastoquinone 9; and UQ-9, ubiquinone 9.

IPP is a prenyl acceptor of the prenyl group originating from DMAPP, in the reaction catalyzed by PTs. IPP and DMAPP, both substrates of PTs, are interconvertible isomers, whose equilibrium is controlled by IPP isomerase (IPPI) [12]. The enzyme is encoded by *IPPI1* (*At3g02780*) and *IPPI2* (*At5g16440*) genes in *A. thaliana*. Both are transcribed into two different protein variants—short with peroxisomal targeting (IPPI1S, IPPI2S), supplying IPP for the cytosolic PTs, and long with either mitochondrial (IPPI1L) or plastidial targeting (IPPI2L), providing IPP for PTs in respective organelles [13,14,15] (Figure 3). In agreement with that, PT activity at the level of a plant cell is also compartmentalized [16].

### 2.1. Trans-Prenyltransferases

C–C double bonds in prenyl-PP can be either in *trans* or *cis* conformation depending on the PT—*trans-*PTs form *trans* isomers, and *cis-*PTs form *cis-*isomers. Considering the length of the produced carbon backbone, *trans-*PTs can be classified as short-chain PTs (C_10–25_), medium-chain PTs (C_30–35_), and long-chain PTs (C_40–50_) [7]. Members of the short-chain ones are in ascending order C_10_ geranyl diphosphate synthase (GPPS), C_15_ farnesyl diphosphate synthase (FPPS), C_20_ geranylgeranyl diphosphate synthase (GGPPS), and C_25_ geranylfarnesyl diphosphate synthase (GFPPS) (Table 1). Their respective products serve as substrates for several downstream enzymes and, as such, represent important branch points in isoprenoid biosynthesis.

**Figure 4:**
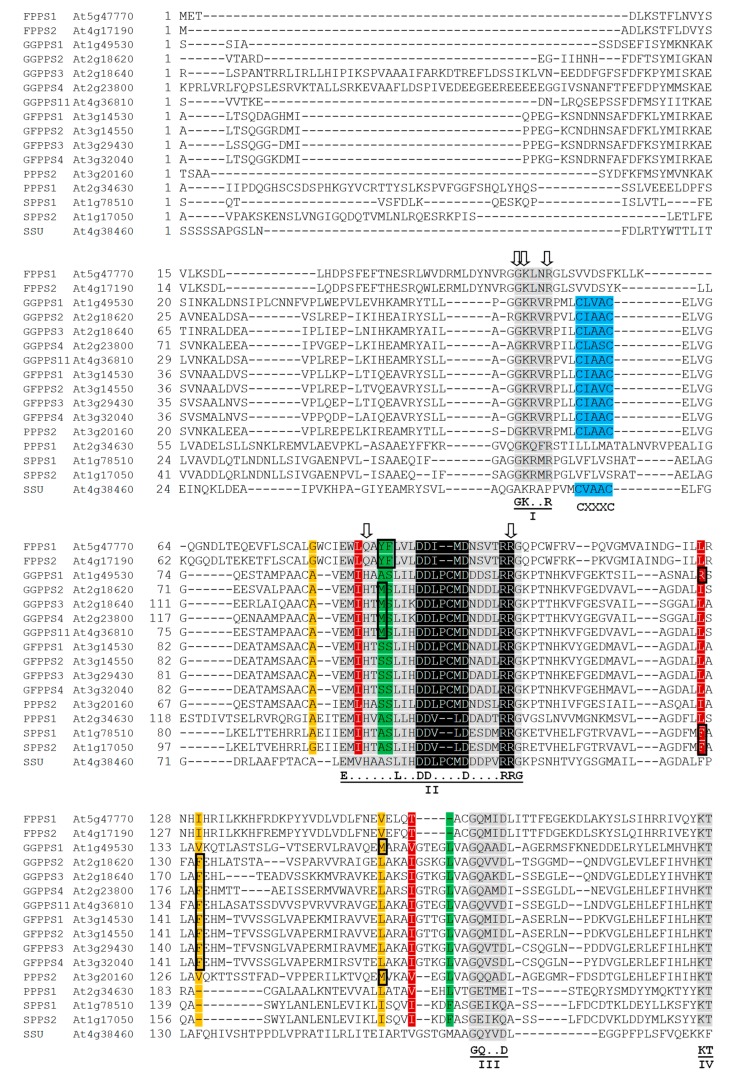

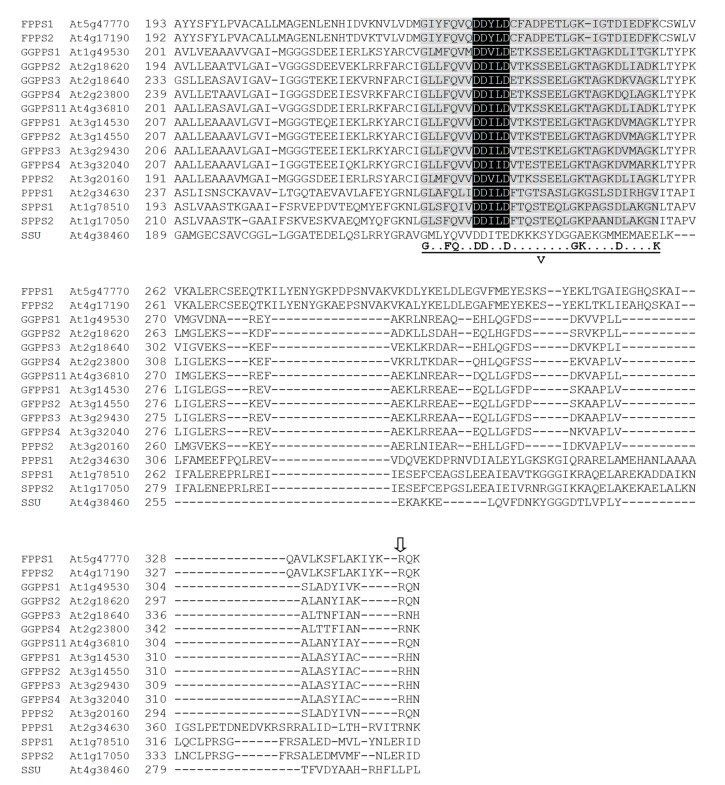
Multiple sequence alignment for *A. thaliana trans-*PTs, listed in Table 1. N-terminal sequences are omitted according to prediction by TargetP-2.0 [21] (http://www.cbs.dtu.dk/services/TargetP). Highly conserved regions are highlighted in gray. Only regions II and III are present in small subunit (SSU). Motifs essential for catalysis and binding of allylic substrate (first aspartate-rich motif (FARM), second aspartate-rich motif (SARM), and +5 and +6 arginines (RR)) are highlighted in black [19,20]. Basal residues active in IPP binding are marked with arrows [22]. The CXXXC motif highlighted in blue is critical for physical interaction between the large subunit (LSU) and the SSU [9]. The floor 1, 2, and 3 residues of the three-floor model [23] are indicated in green, red, and yellow, respectively. Blocking residues are boxed.

C_10_ geranyl diphosphate (GPP), the precursor of monoterpenoids, is the simplest product of the condensation reaction of DMAPP with IPP that is controlled by GPPS residing in plastids [8,17] (Figure 3, Table S1). Initially, the GPPS function was attributed to a homodimeric protein (At2g34630) [18]. Later, however, it was revealed that GPPS in *A. thaliana* exists only as a heterodimer composed of one large subunit (LSU) and one small subunit (SSU) [8,9]. While LSU as a homodimer also functions as GGPPS (GGPPS11, At4g36810), its activity is shifted towards enhanced GPP production, when it dimerazes with SSU (At4g38460). SSU itself is not catalytically active due to the lack of one of the two aspartate-rich motifs, which were shown to play a role in binding prenyl-PP substrates by PTs [19,20] (Figure 4).

For the physical interaction between the subunits, the CXXXC (X is any hydrophobic amino acid residue) motif was found to be critical [9]. This motif is characteristic particularly for the SSU subfamily, but it is also present in GGPP and GFPP synthases and in polyprenyl-PP synthase 2 (PPPS2) (Figure 4). In *A. thaliana*, this type of interaction was reported specifically for GGPPS11 and SSU, earlier annotated as GGPPS12 [24]. Because both enzymes reside in plastids, GPP synthesis can be assigned solely to plastids. This is in agreement with monoterpenoid biosynthesis, which is located in plastids [16].

FPP is formed from DMAPP or GPP and IPP via action of FPPS [25] (Table S1). It is a major substrate for cytosolic and mitochondrial branches of isoprenoid biosynthesis (Figure 3). FPP-derived compounds, such as sterols, brassinosteroids, dolichols, and ubiquinones (UQ), are essential for plant growth and development. Others, such as triterpenoids and sesquiterpenoids, mediate defense reactions. Last, but not least, FPP plays a very important role in protein prenylation and hence in cell signaling [26]. In *A. thaliana*, two *FPPS* genes were identified and characterized—*FPPS1* (*At5g47770*) with two protein variants and *FPPS2* (*At4g17190*) [25,27,28,29,30,31,32]. The longer-variant FPPS1L is targeted to mitochondria, the shorter FPPS1S, together with the FPPS2, is localized to the cytosol. Research on this topic demonstrated that *FPPS* genes are redundant, thus one copy of the gene is enough to keep plants viable with only small changes in metabolite content [25,30,31,32,33]. On the other hand, total loss of FPPS activity is lethal due to severely impaired embryo development [31].

GGPP is a product of GGPPS, which accepts allylic DMAPP, GPP, and FPP in the condensation reaction with IPP [5,9,23] (Table S1). In plant cells, GGPP is used for the synthesis of gibberellins and other diterpenoids, as well as carotenoids; it also participates in chlorophyll, tocopherol, plastoquinone (PQ), UQ, and phylloquinone biosynthesis and enables protein geranylgeranylation (Figure 3). GGPPS is encoded by a gene family in *A. thaliana*. At first, 12 gene paralogs were predicted to form the *GGPPS* gene family (*GGPPS1-12*) [24], but with progressive research in the field and more sophisticated methods of analysis, several gene paralogs were excluded from this family.

One of them was *GGPPS5* (*At3g14510*). Because of a frame-shift mutation in its nucleotide sequence, *GGPPS5* encodes a nonfunctional protein and is therefore considered a pseudogene [17]. SSU has no GGPPS activity on its own, but upon dimerization with GGPPS11, changes its product specificity more towards GPP production [9]. In recent years, all the remaining members of the *GGPPS* family were reexamined for their function, and the analysis brought up surprising results (Table S1). Two independent studies provided evidence that *GGPPS6* (*At3g14530*)*, GGPPS7* (*At3g14550*)*, GGPPS9* (*At3g29430*), and *GGPPS10* (*At3g32040*) encode GFPPS enzymes rather than GGPPS [5,23]. Their names were therefore changed to *GFPPS1*, *GFPPS2*, *GFPPS3*, and *GFPPS4*, respectively [23]. Additionally, GGPPS8 (At3g20160) also failed to synthesize GGPP [5,23] and instead produced a mix of prenyl-PPs ranging from C_20_ to C_40_, with the predominant C_30_–C_35_ species. GGPPS8 was therefore renamed to PPPS2 [23], and together with PPPS1 (At2g34630), represents the medium chain-length PTs in *A. thaliana*. The remaining GGPPS enzymes showed solely or predominantly GGPPS activity and therefore remained members of the *A. thaliana GGPPS* family. However, the GGPPS1 (At1g49530) classification is still controversial, because Nagel et. al. [5] showed that this enzyme has both GFPPS and GGPPS activity, with GFPPS activity being the prominent one, whereas Wang et al. [23] showed that this enzyme has only GGPPS activity (Table S1). Thus, GGPPS1 is considered to be a member of the *GGPPS* gene family, which has five members: mitochondrial GGPPS1, plastidial GGPPS2 (At2g18620), GGPPS11, endoplasmic reticulum (ER)-bound GGPPS3 (At2g18640), and GGPPS4 (At2g23800). Their activities were confirmed in several studies by in vitro enzymatic assays, by genetic complementation of GGPPS-deficient *Escherichia coli*, or by analysis of *A. thaliana* mutants [5,9,17,23,34,36,53,54].

C_25_ GFPP is a precursor of sesterterpenoids, which are compounds with multiple bioactive properties and thus promising pharmacological potential [55]. They were occasionally reported in plants, but their origin had not been addressed until recently. Only with the discovery of GFPPS activity in *A. thaliana* has the topic become more attractive. GFPP is in *A. thaliana* synthesized by GFPPS1 (At3g14530), GFPPS2 (At3g14550), GFPPS3 (At3g29430), and GFPPS4 (At3g32040) (Figure 3). In in vitro tests, these isozymes utilized preferentially GPP, FPP, and GGPP as prenyl donors and, with lower efficiency, DMAPP as well [5,23] (Table S1). Although GFPP is a predominant product of the GFPPSs, the enzymes also have considerable GPPS and GGPPS activity [5,23]. This can explain why complementation tests of carotogenic *E. coli* lacking functional GGPPS provided false positive results when using GFPPS [17,23]. *GFPPS* genes, at least in Brassicaceae, occur in the vicinity of the terpene synthase (*TPS*) genes forming *PT-TPS* clusters [23,56,57].

Long-chain *trans-*PTs in *A. thaliana* are represented by solanesyl diphosphate synthases (SPPSs). Their C_45_ long product solanesyl diphosphate (SPP) can be found in prenylquinones as a prenyl side chain, which serves to anchor the molecule into the membrane [58]. Plant prenylquinones operate in electron transfer during photosynthesis in plastids (PQ) and during respiration in mitochondria (UQ). The length of their prenyl attachments vary among species—in *A. thaliana* plastoquinone 9 (PQ-9) and ubiquinone 9 (UQ-9) are predominant prenylquinone species [45], which means, they have mainly nonaprenyls (C_45_) attached. The first *Arabidopsis SPPS1* (*At1g78510*) gene was described in 2003 by Hirooka et al. [59] The sequence was identified based on the similarity with *Arabidopsis* FPP and GGPP synthases and also bacterial long-chain PTs. According to in vitro enzymatic reaction, SPPS1 could utilize FPP and GGPP but not DMAPP and GPP as substrates. Its predominant product was SPP (Table S1). Another long-chain PT (SPPS2; At1g17050), with 79% amino acid sequence identity to SPPS1 (At1g78510), was described by Hirooka et al. in 2005 [46]. SPPS2, similar to SPPS1, used only FPP or GGPP but not DMAPP with IPP to synthesize SPP (Table S1). In vivo activity of both enzymes was checked in the background of *Schizosaccharomyces pombe dlp1* and *dps1* mutants lacking functional decaprenyl-PP synthase [45]. When either of the mutants expressed *SPPS1* or *SPPS2*, its ability to grow on minimal medium was restored. Both enzymes reside in plastids [42,44,45,46] (Figure 3), although SPPS1 was initially described to localize to the ER [45,46]. In plastids, both enzymes interact with fibrillin FBN5, which is believed to be an indispensable structural component of PQ-9 biosynthesis through the direct stimulation of SPPS activity [44]. On the other hand, FBN5 did not interact with a plastidial fraction of PPPS1 (PPPS1pl; At2g34630), which is a medium-chain polyprenyl-PP synthase also able to catalyze the solanesyl formation [41,43]. PPPS1 was firstly characterized as a homomeric GPPS, active in plastidial monoterpenoid biosynthesis [18]. The corresponding gene shared 24.2%–28.7% identity with *Arabidopsis* short-chain PTs known at that time. When Hirooka et al. studied *SPPS1* [59] and *SPPS2* [46], they noticed a relatively high level of sequence identity between their genes and the previously misidentified *GPPS* (39% and 40%, respectively). However, the information was communicated very marginally, and only later did it draw the needed attention. Hsieh et al. [41] found out that putative GPPS can make C_20_–C_45_ products out of GPP, FPP, or GGPP when IPP is in excess (Table S1). They reasoned that previous in vitro testing of GPPS could have been negatively affected by inappropriate reaction conditions in terms of the used substrates and their mutual ratio. Finally, the enzyme’s participation in UQ biosynthesis was proved by successful complementation of the yeast *coq1* mutant [43]. PPPS1 has dual mitochondrial and plastidial targeting, probably thanks to a genuine bifunctional pre-sequence [60] (Figure 3). In *PPPS1*-silenced and overexpressing *Arabidopsis* lines only the amount of UQ-9 but not PQ-9 was found to vary [43]. Thus, PPPS1 was proposed to synthesize the side chain of UQ-9 in mitochondria but not that of PQ-9 in plastids. Summarizing the data, we can conclude that SPPS1 and SPPS2 contribute to PQ-9 biosynthesis, whereas PPPS1 is involved in UQ-9 biosynthesis. This functional difference is further supported by their separate evolutionary history—SPPS1 and SPPS2 are close relatives of cyanobacterial *trans-*long-chain PTs, whereas PPPS1 has strictly eukaryotic origin [42].

### 2.2. Cis-Prenyltransferases

In plants, *cis-*prenyltransferases (*cis-*PTs or CPTs) catalyze the synthesis of polyprenols and dolichols that are usually longer than C_50_ in length [61]. Because CPTs use short all-*trans-*prenyl diphosphates—mostly FPP and GGPP as priming molecules—which conjugate with IPP in a *cis* orientation (Table S1), the resulting products exhibit mixed di-*trans-*poly-*cis* or tri-*trans-*poly-*cis* stereoisomerism, respectively [62] (Figure 1). While polyprenols are unsaturated at the terminal *α*-unit and are common in bacteria and plant photosynthetic tissues, dolichols are *α*-saturated and typically occur in yeasts, animals, and plant roots [49]. In fact, most organisms accumulate both forms of these polyisoprenoid alcohols, but the composition and ratio vary from species to species and also in a tissue-specific manner. The dynamic nature of polyisoprenoid metabolism can be observed during development or in response to various environmental stimuli suggesting diverse biological functions of these compounds [47]. Polyprenols, as well as dolichols, have the capacity to modulate the properties of membranes, they can serve as donors for protein prenylation, and dolichols, in particular, are inevitable saccharide carriers in protein glycosylation. Moreover, polyprenols that are quite abundant in chloroplasts can affect photosynthetic efficiency [51]. Current knowledge on the polyisoprenoid synthesis and function in plant cells is rather restricted and regulatory mechanisms controlling their biosynthesis are far from being understood.

Searching for plant *CPTs*, nine yeast *CPT* homologues (*CPT1-9*) and gene-encoding CPT-binding protein (CBP) *LEW1* were identified in the *A. thaliana* genome (Table 1). So far, only four CPTs have been characterized and proven to be functional CPT enzymes. The first *Arabidopsis* CPT (CPT1, At2g23410) was described by the independent studies of Oh et al. [63] and Cunillera et al. [64]. Interestingly, the protein produced a different spectrum of polyprenols in vitro and in planta (more than C_100_) [49,63] and in vivo using *rer2Δ* yeasts that lack intrinsic CPT activity (less than C_100_) [49,64]. Moreover, the range of detected polyisoprenoids differed slightly even between roots and leaves in *Arabidopsis CPT1* overexpressing lines [49]. Species and tissue-specific differences in chain length that were observed may be the result of different regulation of polyisoprenoid biosynthesis in different species and tissue background or may result from the availability of different substrates. Biosynthesis of short-chain polyisoprenoids in *A. thaliana* is mediated by heptaprenyl-PP synthase CPT6/HEPS (At5g58780), which catalyzes the formation of dolichol with seven isoprenoid units (Dol-7) [48,50] (Table S1). Both CPT1 [49] and CPT6 [48,50] were found to reside in the ER (Figure 3). CPT7 (At5g58770) is so far the only plastidial CPT that was characterized in *Arabidopsis* [51] (Figure 3). Following its subcellular localization, GGPP not FPP is likely the substrate for the synthesis of C_55_ polyisoprenoids, and mutant analysis of the *cpt7-1* null mutant showed that CPT7 influences photosynthetic performance via modulation of thylakoid membrane dynamics.

There are two types of CPTs in plants—homomeric and heteromeric [65]. Homomeric ones act autonomously in the contrary to the heteromeric CPT enzymes, which need to dimerize with a CBP to gain its activity [66,67,68]. So far, the only CBP identified in *A. thaliana* is LEW1 (At1g11755), which was described to form a heterocomplex with CPT3 (At2g17570). It was shown that CPT3/LEW1 synthesizes long-chain polyisoprenoids, whereas individual CPT3 or LEW1 proteins were catalytically inactive [10]. Synthesis of polyisoprenoids by CPT/CBP heterocomplexes is typical for the eukaryotic lineage of CPTs, based on the phylogenetic analysis. Besides CPT3 (At2g17570), CPT4 (At5g60510) and CPT5 (At5g60500) also belong to this lineage in *A. thaliana* [48]. The remaining *Arabidopsis* CPTs cluster together in a prokaryotic clade, suggesting that they are self-sufficient homomeric enzymes. Recently, it was shown, that CBPs and homomeric CPTs have in common a highly conserved RXG (eventually NXG) motif in their C-terminal sequence, which is essential for catalysis [65] (Figure S1). On the other hand, the motif is absent in the heteromeric CPTs, and therefore, interaction with a CBP is required. Furthermore, CBPs are not able to bind substrates due to deletion, and thus, creating a CPT/CBP dimer is essential for the function of both proteins.

## 3. Primary Structure of PTs Can Be Indicative for Determination of Their Function

Protein functions are closely related to their structures. However, the structure was determined only for a small fraction of the myriad protein sequences deposited to databases. Therefore, functional characterization of novel sequences still heavily relies on homology-based approaches, in which experimentally determined functions are transferred to uncharacterized homologous sequences with various levels of confidence [69]. Such methods use the fact that homologous sequences sharing a high level of identity (at least 40%) and similarity are often isofunctional. However, *Arabidopsis* GGPP and GFPP synthases are functionally different, although they share more than 55% sequence identity [23]. Thus, the sequence alignment is not robust enough to assign the function to a protein, although it is still a convenient method for rough classification of unknown proteins.

Five highly conserved regions (I–V) can be recognized in the sequence of every *trans-*PT [70] (Figure 4). Regions II and V include aspartate-rich motifs DDX_(2-4)_D, referred to as the first aspartate-rich motif (FARM) and the second aspartate-rich motif (SARM). These motifs and +5 and +6 arginines (RR) downstream from the FARM are essential for catalysis and binding of the allylic substrate [19,20]. The attachment of IPP is coordinated by the C terminus of the enzyme and dispersed conserved basal residues [22]. From the point of the tertiary structure, all conserved features are associated with the active site, which is located in a large deep cleft of the enzyme formed out solely of *α-*helices [23,41]. The capacity of the catalytic pocket, where prenyl-PP elongation takes place, is determined by residues, which protrude into the cavity. Residues with large side chains can obstruct the cavity and thus further prenyl elongation [71]. It is currently unknown whether the mechanism is also valid for CPTs.

To describe the residues, which are an obstacle in the enzyme elongation channel, several authors have adopted the term “floor”. So far, single-, double-, and three-floor models of *trans-*PT chain-length determination have been proposed [23,41,71,72,73,74]. The three-floor model suggested by Wang et al. [23] is the most precise one. The model was deduced from the observations made on the crystal structures of three representatives of *Arabidopsis* PTs—GGPPS11, GFPPS2, and PPPS2. The authors revealed that some residues within the enzyme pocket assemble to form a floor, and there are three such floors along the pocket and thus three potential points where the synthesis of a prenyl product can be arrested due to steric hindrance from the blocking residue(s). Following the model scheme, the FPPS activity can be predicted from the two bulky residues (Tyr and Phe) on floor 1 [71] (Figure 4).

The GGPPS activity could be predicted from Phe on floor 3 and medium-sized Met on floor 1, which only partially closes the cavity (Figure 4). If solely Phe on floor 3 is present, the reaction should terminate with the release of GFPP (Figure 4). Dual GGPP/GFPP function could be indicated by Arg on floor 2 and Met on floor 3, which were detected in GGPPS1 (Figure 4). Finally, PPPS activity could be deduced from the lack of blocking residues, because there is only Met on floor 3 in PPPS2 (Figure 4). Predictions using the three-floor model are in agreement with experimental data on the product specificity of selected enzymes; thus, the model is functional at least with *Arabidopsis* short- and medium-chain *trans-*PTs. However, it would be interesting to see if it also works with long-chain *trans-*PTs. Mapping the model’s sites on the respective residues in PPPS1 has shown that no bulky residue is present in the pocket, but there are two in the SPPS1 and SPPS2—two molecules of Phe on floor 1 and 2 (Figure 4). Following the concept of the three floors model, the lack of obstacles in PPPS1 suggests that it is indeed PPPS. On the other hand, two residues blocking the cavity in SPPS are rather unlikely and hence challenge the relevance of the model with respect to the prediction of long-chain *trans-*PTs. In that case, the crystal structure of SPPS would be needed to optimize the model, but so far it is not available. Yet, we can see that in case of PTs, not the total sequence similarity but either the presence of specific domains or amino acid residues have predictive power when analyzing the amino acid sequence.

In past decades, determination of the key residues forming the pocket had been preceded by resolving the three-dimensional (3D) structure of the enzyme and/or laborious site-directed mutagenesis experiments [22,23,41,73,74,75,76]. In recent years, however, we can witness considerable efforts to create in silico prediction models, which could reliably assign function to a protein based on its raw sequence [1,77,78]. Naturally, 3D conformations remain an invaluable source of data needed to design a powerful algorithm.

## 4. Prenyltransferase Mutants: A Source of Information about Their Function and Topology

Analysis of knockouts and knockdowns is the way to understand the functionality of pathway enzymes within an in planta context. Visible phenotypes, moreover, can have predictive function for the topology of yet uncharacterized enzymes and for our knowledge on carbon fluxes via the isoprenoid pathway network. Both specific inhibitors and gene mutants were extensively used to analyze early enzymatic steps in the isoprenoid pathway (MVA and MEP pathways). In the case of PTs, however, mainly mutants were used to characterize their in planta function (Table 2).

Mutants in the linear metabolic pathway are expected to have similar phenotypes. MVA pathway null mutants are embryo lethal or male sterile, affected in male gametophyte development or in pollen tube elongation, whereas MEP pathway mutants grow to the seedling stage, but remain albinos [2]. In fact, there is only one null pathway mutant in the MVA pathway that is embryo and not male gametophyte lethal. It is an *aact2* mutant impaired in acetoacetyl-CoA transferase, acting at the beginning of the MVA pathway [79]. It is expected that mutants in essential downstream pathways stemming from either MVA or MEP pathway will have identical or similar phenotypes. Indeed, mutants in the cytosolic/mitochondrial essential pathways, such as the sterol biosynthetic pathway, UQ biosynthetic pathway, protein Rab geranylgeranylation pathway, and dolichol biosynthetic pathway, have either impaired male gametophyte function or are embryo lethal [80,81,82,83]. Similarly, mutants in essential biosynthetic plastidial pathways, such as the carotenoid biosynthetic pathway, chlorophyll biosynthetic pathway, and PQ biosynthetic pathway, are albino or pale green [36,42,84,85]. In agreement with that, mutants in PTs that provide substrates for those specialized isoprenoid pathways should display similar phenotypes.

### 4.1. Prenyltransferase Mutants in the Cytosolic/Mitochondrial Branch of the Isoprenoid Pathway

The first step in the DMAPP and IPP condensation in the cytosol and mitochondria is FPP synthesis (Figure 3). There are two enzymes (FPPS1S, FPPS2) in the cytosol and one enzyme (FPPS1L) in the mitochondria that synthesize FPP. FPPS1L is encoded by the same locus as FPPS1S but only has mitochondrial targeting sequence. Neither the *fpps1* nor the *fpps2* null mutant (the *fpps1* mutant is mutant in both FPPS1S and FPPS1L enzymes) shows the visible phenotype, and the levels of sterols and UQ-9, the major cytosolic and mitochondrial isoprenoids, are only slightly reduced in single mutants (Table 2). This demonstrates that both enzymes are highly redundant in *Arabidopsis*. Moreover, it shows that the function of FPPS1L in the mitochondria is not essential, excluding FPPS1L from being the only source of substrate for UQ-9 biosynthesis.

The *fpps1fpps2* double mutant is embryo lethal, arrested at the globular stage of development. The *fpps1fpps2* male gametophyte is only slightly affected in pollen tube elongation, which means that compared with MVA pathway mutants, which are mostly male gametophyte lethal, the *fpps1fpps2* phenotype is less severe [2,31]. FPP is in the cytosol mainly used for the synthesis of phytosterols and the null mutant in the cycloartenol synthase (*cas1*), which synthesizes cycloartenol for the phytosterol biosynthetic pathway, is also male gametophyte lethal, as most of the MVA pathway mutants [81]. The less severe phenotype of *fpps1fpps2* as compared with other mutants in sterol biosynthesis indicates that FPPS activity can be partly complemented, at least in some developmental stages. So far, we can only speculate about the source of the complementation. FPP can be imported from the wild-type cells of surrounding tissues or be synthesized by other PTs. For example, GGPPS11, which is also present in the cytosol, can synthesize a small amount of FPP (2.1%) [9] (Table S1).

The second step in DMAPP and IPP condensation in the cytosol and mitochondria is GGPP synthesis (Figure 3). There are three GGPPS enzymes in the cytosol (GGPPS3, GGPPS4, and GGPPS11S) and one in the mitochondria (GGPPS1). *GGPPS11* transcript is, among all the *GGPPS* paralogs, the most abundant and the only one ubiquitously expressed in plants [17]. So far, *ggpps11* and *ggpps1* mutants have been isolated and characterized (Table 2). *ggpps1* has no visible phenotype and no decrease in metabolite levels, including UQ-9 [36]. On the contrary, the *ggpps11* mutant showed embryo lethal phenotype with the embryo arrested at the heart stage of development [36,54]. A similar phenotype is typical for the mutants deficient in the UQ biosynthesis [80]. The level of UQ-9 was tested in the *ggpps11-5* knockdown mutant, which has decreased *GGPPS11* transcript levels to about 50%. In this mutant, the level of UQ-9 was slightly decreased but not significantly [36], suggesting that the lack of UQ-9 is not the (main) reason for the embryo lethal phenotype of *ggpps11* and suggesting, together with the results of *ggpps1* analysis, that FPP rather than GGPP could be the source of prenyl-PP substrate for the UQ biosynthesis.

The level of UQ-9 was decreased significantly in *fpps1* and *fpps2* single mutants [31] and *FPPS*-silenced plants [32]. However, approximately 50% decrease in total FPPS activity, at the 16% level of transcripts for *FPPS1* and 35% for *FPPS2* in *FPPS*-silenced plants, caused only 30% decrease in UQ-9 levels [32]. This suggests that although FPPS1 and FPPS2 are contributing significantly to UQ biosynthesis, they are not the only substrates for SPP and subsequently UQ-9 biosynthesis. C_45_ solanesyl moiety, the predominant form of UQ prenyl chains in *Arabidopsis,* is synthesized by a mitochondrial PPPS1 (PPPS1mt) that elongates an initial FPP or GGPP with IPP units [41,43]. The *ppps1* null mutant is, similarly to other UQ biosynthetic mutants, embryo lethal [80,87]. The reduction of *PPPS1* transcript levels by 80% in *PPPS1*-silenced plants caused almost proportional reduction in UQ-9 content (reduction by 75–80%) [43]. This indicates that while PPPS1 is the only enzyme synthesizing C_45_ for UQ-9 biosynthesis, FPPS and GGPPS will have a rather promiscuous function in this process.

The question of whether the lack of GGPPS activity in the cytosol and mitochondria can to some degree be complemented by other sources of GGPP cannot be answered at the moment, because we do not have mutant(s) with null GGPPS activity in both the cytosol and mitochondria. What we can say so far is that the *ggpps11* mutant has a less severe phenotype than the Rab geranylgeranylation null mutant [82]. A complete lack of Rab prenylation, which uses GGPP as a substrate, causes male sterility.

So far, in *Arabidopsis*, mutants in three genes synthesizing polyprenols in the cytosol and/or mitochondria were characterized, *cpt1*, *cpt6*, and *lew1* (Table 2). In all three mutants, dolichol levels were reduced, but in each of them, different classes of dolichols were affected. While synthesis of long chain dolichols was reduced in the *cpt1* and *lew1* mutants [49], short chain dolichols were reduced in the *cpt6* mutant [50]. The phenotype was also very different in all the mutants. No visible phenotype was reported for *cpt6* [50], *cpt1* null mutation affected plant growth and root elongation [49], and *lew1* null mutation is lethal, although it is not known whether it is due to the embryo lethality or gametophyte lethality [88]. Mutants in later steps of dolichol biosynthesis, in polyprenol reductase (PPRD2), which catalyzes the conversion of polyprenol to dolichol, affects male gametophyte development and pollen germination [83]. Thus, the function of different CPTs in plant dolichol synthesis within the context of plant physiological processes still remains largely unknown.

### 4.2. Prenyltransferase Mutants in the Plastidial Branch of the Isoprenoid Pathway

The first product of DMAPP and IPP condensation in plastids is GPP, which in *Arabidopsis* is synthesized by the heteromeric enzyme GGPPS11/SSU (Figure 3). In agreement with that, both the knockdown *ggpps11* mutant and the null *ssu* mutant have reduced levels of monoterpenoids to approximately 50% [11] (Table 2). Heterodimer GGPPS11/SSU is so far considered to be the only GPPS in *Arabidopsis*. Nevertheless, because monoterpenoids are still synthesized in *Arabidopsis* in the *ssu* knockout mutant, it is likely that other GPP-synthesizing enzymes are active in *Arabidopsis*. In addition to GGPPS11, small GPPS activity was also observed in GFPPSs [5,23] (Table S1). Previously, PPPS1 was classified as GPPS, because it was shown to synthesize GPP predominantly and was assumed to function in monoterpenoid biosynthesis and gibberellin biosynthesis in plastids [18,87]. Nevertheless, *PPPS1*-RNAi plants do not have a decreased level of monoterpenoids [8], and GPPS was shown to have PPPS (C_20_–C_45_) activity [41] (Table S1).

In agreement with the proposed housekeeping role of GGPPS11 [17,54], the *ggpps11* knockdown mutant has lower levels of chlorophylls, carotenoids, phylloquinones, PQs, and tocopherols [8,36,54]. In addition to GGPPS11, which is the main GGPP-synthesizing enzyme in plastids and essential for the synthesis of photosynthesis-related isoprenoid compounds [54], GGPPS2 is another GGPPS paralog in plastids (Figure 3). The *ggpps2* null mutant shows no visible developmental defects, and the level of carotenoids, chlorophylls, and prenylquinones is at the wild-type level (Table 2), demonstrating that GGPPS2 does not contribute significantly to the synthesis of photosynthesis-related plastidial isoprenoids that are essential for growth and development, and therefore, the function of this isozyme is yet to be determined [54].

The newly discovered group of PTs, GFPP synthases (GFPPS1-4) that synthesize GFPP, resides in plastids [17] (Figure 3). GFPPSs predominantly synthesize GFPP, which is a precursor of sesterterpenoids [5,23]. There are four GFPPSs in *Arabidopsis* plastids. GFPPS null mutants *gfpps1*, *2*, *4* and *GFPPS3*-RNAi lines with 21% and 16% of mRNA levels, respectively, show no visible phenotypes [54] (Table 2).

*Arabidopsis* possesses two paralogous SPP synthases, SPPS1 and SPPS2, which assemble the side chain of PQ-9 in plastids (Figure 3). The *spps1spps2* double knockout is devoid of PQ-9 and plastochromanol and cannot grow photoautotrophically [42] (Table 2). Because the preferred substrate for SPPS is GGPP and not DMAPP or GPP [59] (Table S1) and the *ggpps11* mutant has decreased PQ-9 and plastochromanol levels [54], we can predict that the main enzyme generating substrate for SPPS in plastids is GGPPS11.

PPPS responsible for the synthesis of prenyl chain of UQ-9 in the mitochondria is also localized in plastids (PPPS1pl) because of double targeting [18,43] (Figure 3). While PPPS1mt synthesizes the side chain of UQ, PPPS1pl is not involved in PQ biosynthesis [43], and the function of PPPS1pl thus remains to be discovered. In tomato, downregulation of *PPPS1* transcript led to a decreased level of gibberellins [87], but it was not tested whether PPPS1pl has the same function in *Arabidopsis*.

There is another PPPS in plastids, PPPS2, which synthesizes carbon chains of C_20–40_ [23] (Figure 3, Table S1). The null *ppps2* mutant has no visible phenotype, and from all photosynthetically related metabolites that were analyzed, the level of chlorophyll b was slightly decreased and levels of *β*-carotene and neoxanthin slightly increased. The levels of other metabolites, such as chlorophyll a and phylloquinone were unchanged [54] (Table 2).

There is one CPT predicted to localize to plastids (Figure 3). The null mutant in plastidial CPT7 has a decreased level of plastidial polyprenols (C_45_–C_55_ with a maximum of C_50_) by 100% (Table 2). Affected plants have impaired photosystem II operating efficiency, and their thylakoids exhibit a decreased rate of electron transport but are not lethal [51].

## 5. Co-Expression Analysis and Prediction of Isoprenoid Fluxes

Co-expression analysis determines clusters of genes that are co-regulated together at expression levels. Metabolic pathway genes involved in the same physiological process are often co-regulated, which was also shown for genes in the isoprenoid pathway [89,90,91]. Therefore, gene association analysis based on the gene co-expression can be very instrumental in uncovering novel metabolic pathway genes or in determining their topology in the pathway gene network.

There are several examples where gene co-expression analysis resulted in better understanding of the prenyl-PP synthase function. For example, co-expression analysis with plastidial GGPPS and GFPPS isozymes showed that only *GGPPS11*, which is the ubiquitously expressed plastidial *GGPPS* paralog and the only one highly expressed in green tissues [17], is significantly co-expressed with all genes encoding MEP pathway enzymes and almost all genes from pathways synthesizing photosynthesis-related isoprenoids. The result of this co-expression analysis was also supported by the metabolite profiling of *ggpps11* mutant [54] (Table 2). Co-expression analysis also correctly predicted subcellular localization of SPPS1 in plastids [42], although previous experimental evidence showed that this protein is associated with the ER [45,46]. This prompted further experiments that proved that *SPPS1* is a paralog of *SPPS2* and both enzymes are responsible for the plastidial synthesis of SPP and are essential for PQ and plastochromanol biosynthesis [42]. Co-expression analysis was also instrumental in assigning function to the PPPS1 protein. The PPPS1 protein was originally annotated as GPPS. It was shown that GPPS produces GPP in vitro, and GFP fusion of the protein (GPPS-GFP) localizes to plastids [18]. Nevertheless, the embryo lethal phenotype of the knockout mutant was rather nontypical for a plastidial isoprenoid pathway enzyme. In search of *Arabidopsis* isoprenoid pathway genes that are co-expressed with the *Arabidopsis* Ubi/Coq orthologs (UQ biosynthesis genes), GPPS was surprisingly found to be associated with the gene co-expression cluster. The enzymatic function of GPPS was reevaluated, and it was shown that GPPS actually synthesizes PPPs of different length (C_25_–C_45_), with C_35_ being the predominant prenyl-PP, and the enzyme was renamed to be PPP synthase 1 (PPPS1) [41,43]. Localization experiments using the expression of the GFP fusion protein driven by the native promoter have shown that PPPS1 localizes both to plastids and to the mitochondria [43].

We applied the same co-expression approach, which we used earlier for the plastidial *GGPPS* and *GFPPS* gene paralogs [54], to short *trans*-PTs to see whether we can extract additional information as to the function of PTs within the isoprenoid pathway gene network (Table 3, Table S2). Microarray expression data from different *A. thaliana* organs and tissues were used for analysis as described in Table S2. In the cytosol, as expected, *FPPS1* and *FPPS2* genes are the most co-expressed with the genes from the MVA and sterol biosynthetic pathways and both to the same extent supporting functional redundancy of both enzymes. As to the other functionally uncharacterized PTs and their involvement in the biosynthetic pathways in the cytosol and mitochondria, we can see that both *FPPS* and *GGPPS* genes and gene paralogs have connections to the genes of the UQ biosynthetic pathway and to the protein prenylation, and there is likely no strict allocation of a single *FPPS* or *GGPPS* gene paralog to those pathways. This seems not to be true for the diterpenoid biosynthesis, where *GGPPS3* has the most gene connections to *TPSs*. In plastids, the synthesis of isoprenoid compounds associated with photosynthesis is associated with the single *GGPPS11* gene paralog, as observed previously [54], except in the phylloquinone biosynthetic pathway, which is here, with the current gene expression set, more connected to *GGPPS2* and other *PTs*. Genes synthesizing terpenoids and gibberellins are also instead associated with several isoprenoid synthases and their paralogs. Surprisingly, monoterpenoids show more connections to *PPPS1*, which was previously annotated as *GPPS*, than with *SSU*.

Metabolic gene clusters within dynamic chromosomal regions could provide another mechanism of transcriptional co-regulation. It has become clear in recent years that many plant secondary metabolic pathways or some of their genes are clustered [92]. These clusters have arisen by recruitment of genes from elsewhere in the genome through duplication and neofunctionalization. *GFPPS–TPS* gene clusters, where *GFPPS* genes and *TPS* genes are in close proximity were also found in *Arabidopsis* [23,56,57].

## 6. Conclusions

In the last decade, we have witnessed enormous progress in the characterization of *Arabidopsis* isoprenoid pathway PTs. Enzymes that were initially defined only by their approximate enzymatic function are now more precisely characterized at the enzymatic level, and their biological role, as well as topology, within the isoprenoid pathway network is clearly defined. PTs with essential function in the cytosol, mitochondria, and plastids are known as metabolic processes controlled by those PTs. What still remains as a challenge is to define at the gene level metabolic routes (fluxes) between PTs and TPSs and between PTs and pathways where prenyl-PP as a substrate is ambiguous; to understand minor, dynamic, developmentally controlled fluxes; and, last but not least, to explore more physiological functions of *cis*-PTs.

## Figures and Tables

**Table 1:** Enzymes and gene models of prenyltransferases in *A. thaliana*.

ENZYME			GENE						
Acronym	Name	EC Number	AGI	Gene Model	References	Alternative Names	References	Localization ^b^	References
GPPS	Geranyl diphosphate synthase **^c^**	EC 2.5.1.1	*At4g36810/At4g38460*	*GGPPS11L**^a^**/SSU*		*IDS11; GGPPS11; GGPS1/GGPPS12*	[5,24,34]/[24]	Pl/Pl	[17,34,35,36]/[8]
FPPS	Farnesyl diphosphate synthase	EC 2.5.1.10	*At5g47770*	*FPPS1S**^a^***	[24,37]			C	[25]
				*FPPS1L**^a^***	[24,37]			Mt	[37]
			*At4g17190*	*FPPS2*	[24]			C	[25]
GGPPS	Geranylgeranyl diphosphate synthase	EC 2.5.1.29	*At1g49530*	*GGPPS1*	[24]	*IDS1; GGPS6*	[5,34]	Mt	[5,17,34,35,38]
			*At2g18620*	*GGPPS2*	[24]	*IDS2*	[5]	Pl	[17]
			*At2g18640*	*GGPPS3*	[24]	*IDS3; GGPS4*	[5,34]	ER	[17,34]
			*At2g23800*	*GGPPS4*	[24]	*IDS4; GGPS2*	[5,34]	ER	[17,34,39]
			*At4g36810*	*GGPPS11S**^a^***	[36]			C	[36,40]
			*At4g36810*	*GGPPS11L**^a^***		*IDS11; GGPPS11; GGPS1*	[5,24,34]	Pl	[17,34,35]
GFPPS	Geranylfarnesyl diphosphate synthase	EC 2.5.1.81	*At3g14530*	*GFPPS1*	[23]	*IDS6; GGPPS6*	[5,24]	Pl	[5,17]
			*At3g14550*	*GFPPS2*	[23]	*IDS7; GGPPS7; GGPS3*	[5,24,34]	Pl	[5,17,34]
			*At3g29430*	*GFPPS3*	[23]	*IDS9; GGPPS9*	[5,24]	Pl	[5,17]
			*At3g32040*	*GFPPS4*	[23]	*IDS10; GGPPS10*	[5,24]	Pl	[5,17]
PPPS	Polyprenyl diphosphate synthase	EC 2.5.1.91	*At2g34630*	*PPPS1*	[41]	*GPPS; SPPS3*	[24,42]	Pl/Mt	[18,43]
			*At3g20160*	*PPPS2*	[23]	*IDS8; GGPPS8*	[5,24]	Pl	[17]
SPPS	Solanesyl diphosphate synthase	EC 2.5.1.85	*At1g78510*	*SPPS1*	[42]			Pl	[42,44]
			*At1g17050*	*SPPS2*	[42]			Pl	[44,45,46]
CPT	*cis*-Prenyltransferase	EC 2.5.1.87	*At1g11755*	*LEW1*	[47]			PM***^d^***	
			*At2g23410*	*CPT1*	[47]	*AtcPT3*	[48]	ER	[48,49]
			*At2g23400*	*CPT2*	[47]	*AtcPT2*	[48]	C***^d^***	
			*At2g17570*	*CPT3*	[47]	*AtcPT1*	[48]	Mt***^d^***	
			*At5g60510*	*CPT4*	[47]	*AtcPT9*	[48]	C***^d^***	
			*At5g60500*	*CPT5*	[47]	*AtcPT8*	[48]	C***^d^***	
			*At5g58780*	*CPT6*	[47]	*AtcPT5*	[48]	ER	[48,50]
			*At5g58770*	*CPT7*	[47]	*AtcPT4/AtHEPS*	[48,51]	Pl	[48,51]
			*At5g58782*	*CPT8*	[47]	*AtcPT6*	[48]	ER	[47]
			*At5g58784*	*CPT9*	[47]	*AtcPT7*	[48]	ER***^d^***	

Abbreviations: EC, Enzyme Commission; AGI, The *Arabidopsis* Genome Initiative. ^a ^Alternative transcription start site. ^b^ Subcellular localization: C, cytosol; ER, endoplasmic reticulum; Mt, mitochondrion; Pl, plastid; PM, plasma membrane. ^c ^Heterodimeric GPP synthase. ^d ^SUBA prediction [52]; http://suba.live/aboutSUBA4.html.

**Table 2:** Mutations in prenyltransferase genes and their phenotypes in *A. thaliana*.

Gene	AGI	Mutagen	Allele	Mutant Line	Phenotype	References
*FPPS1*	*At5g47770*	T-DNA	*fpps1-1*	SAIL_310-D07	Wild-type phenotype with slightly reduced levels of sterols and UQ-9	[31]
		T-DNA	*fpps1-2*	SALK_073576		
*FPPS2*	*At4g17190*	T-DNA	*fpps2-1*	SAIL_328_G06	Wild-type phenotype with slightly reduced levels of sterols and UQ-9	[31]
		Ds	*fpps2-2*	GT7041		
*FPPS1/FPPS2*	*At5g47770/At4g17190*	T-DNA/Ds	*fpps1/fpps2*	SAIL_310_D07; SALK_073576/SAIL_328_G06; GT7041	Embryo lethal at the globular stage; slightly impaired pollen tube elongation	[31]
		amiRNA	*fpps1/fpps2*	amiFPS1 (21%/26% mRNA), amiFPS2 (16%/35% mRNA)	Impaired growth and development; chlorosis; reduces level of chlorophylls, carotenoids, sterols and UQ-9; higher level of UQ-10	[32]
*SSU*	*At4g38460*	T-DNA	*ssu-1 (ggpps12-1)*	pst11416	Wild-type phenotype with reduced level of monoterpenoids, and wild-type level of carotenoids, chlorophylls, sesquiterpenoids	[8]
		T-DNA	*ssu (ggpps12)*	pst01798 (20% mRNA)		
*GGPPS1*	*At1g49530*	T-DNA	*ggpps1-1*	SAIL_559_G01	Wild-type phenotype with wild-type level of UQ-9, carotenoids, tocopherols, chlorophylls, PQ-9, phylloquinones, plastochromanol-8	[36]
*GGPPS2*	*At2g18620*	T-DNA	*ggpps2-1*	FLAG_134_B10	Wild-type phenotype with wild-type levels of carotenoids, chlorophylls, phylloquinones	[54]
*GGPPS3*	*At2g18640*	-	-	-	-	-
*GGPPS4*	*At2g23800*	-	-	-	-	-
*GGPPS11*	*At4g36810*	EMS mutagenesis	*ggpps11-1*	D163R point mutation	Variegated phenotype; germination delayed on the inhibitor of gibberellin biosynthesis; reduced level of chlorophylls and carotenoids	[54,86]
		T-DNA in chloroplast targeting sequence	*ggpps11-2*	SALK_015098	Albino seedling	[54,86]
		T-DNA	*ggpps11-3*	SALK_085914	Embryo lethal at the heart stage	[54,86]
		T-DNA	*ggpps11-4*	SAIL_712_D06		[54]
		T-DNA in 5′-UTR	*ggpps11-5*	SALK_140601	Pale green phenotype with reduced level of monoterpenoids, carotenoids, tocopherols, chlorophylls, PQ-9, phylloquinones, plastochromanol-8	[8,36,54]
*GFPPS1*	*At3g14530*	T-DNA	*gfpps1-1 (ggpps6-1)*	SAIL_1148_A03	Wild-type phenotype with wild-type level of carotenoids, chlorophylls, phylloquinones	[54]
*GFPPS2*	*At3g14550*	T-DNA	*gfpps2-1 (ggpps7-1)*	SALK_119280	Wild-type phenotype with wild-type level of carotenoids, chlorophylls, phylloquinones	[54]
*GFPPS3*	*At3g29430*	RNAi	*gfpps3 (ggpps9)*	RNAi GGPPS9-1 (21% mRNA); RNAi GGPPS9-6 (16% mRNA)	Wild-type phenotype with wild-type level of carotenoids, chlorophylls, phylloquinones	[54]
*GFPPS4*	*At3g32040*	T-DNA	*gfpps4-1 (ggpps10-1)*	SM_3_32015	Wild-type phenotypes with wild-type level of carotenoids, chlorophylls, phylloquinones	[54]
*PPPS1*	*At2g34630*	T-DNA	*ppps1-1 (gps1-1)*	GABI 097_G02	Embryo lethal	[87]
		RNAi	*ppps1*	RNAi PPPS1-1-6 (10% mRNA)	Growth reduction; dwarfed plants with delayed flowering	[87]
		RNAi	*ppps1*	RNAi PPPS1-1-3 (20% mRNA)	Reduced level of UQ-9; wild-type level of PQ	[43]
		RNAi	*ppps1*	GPPS RNAi-2, GPPS RNAi-4 (10% mRNA)	Wild-type phenotype with wild-type level of monoterpenoids and sesquiterpenoids	[8]
*PPPS2*	*At3g20160*	T-DNA	*ppps2-1 (ggpps8-1)*	FLAG_470_E09	Higher level of carotenoids; reduced level of chlorophyll b	[54]
*SPPS1*	*At1g78510*	T-DNA	*spps1*	SALK_126948	Wild-type phenotype with reduced level of PQ and plastochromanol, and wild-type level of tocopherol and UQ	[42]
*SPPS2*	*At1g17050*	T-DNA	*spps2*	SALK_064292	Developmental delay; stunted phenotype and pale yellowish leaves at the high light; reduced level of PQ; no plastochromanol; higher level of tocopherol, and wild-type level of UQ	[42]
*SPPS1/SPPS2*	*At1g78510/*	T-DNA	*spps1/spps2*	SALK_126948/SALK_064292	Seedling lethal, albino phenotype; no PQ and plastochromanol; wild-type level of UQ and tocopherol	[42]
	*At1g17050*					
*LEW1*	*At1g11755*	EMS mutagenesis	*lew1-1*	G159A point mutation	Leaf-wilting phenotype, increased drought resistance, impaired plasma membrane integrity, impaired protein *N*-glycosylation, reduced the total plant content of main dolichols C_75_, C_80_ by 85% and protein glycosylation defects	[88]
		T-DNA	*lew1-2*	SALK_032276	Lethal	[88]
*CPT1*	*At2g23410*	T-DNA	*cpt1-1*	SALK_038151	Extremely stunted growth and shorter root length; reduced level from Dol-18 to -23 (Dol-21 dominating)	[49]
		T-DNA	*cpt1-2*	SALK_032276		
		T-DNA	*cpt1-3*	SALK_100795		
*CPT2*	*At2g23400*	-	-	-	-	-
*CPT3*	*At2g17570*	-	-	-	-	-
*CPT4*	*At5g60510*	-	-	-	-	-
*CPT5*	*At5g60500*	-	-	-	-	-
*CPT6*	*At5g58780*	T-DNA	*cpt6-1*	SALK_071255	Reduced level of Dol-7 and short-chain dolichols (Dol-13 dominating)	[50]
			*cpt6-2*	SALK_064499		
*CPT7*	*At5g58770*	T-DNA	*cpt7-1*	SALK_022111	Decreased thylakoid membrane fluidity and photosynthetic performance; no polyprenols 9, 10, 11 (Pol-10 dominating); wild-type level of tocopherols, phylloquinone, carotenoids, PQ, and chlorophylls	[51]
		RNAi	*cpt7*	RNAi-23; RNAi-24 and RNAi-31 (15% mRNA)		
*CPT8*	*At5g58782*	-	-	-	-	-
*CPT9*	*At5g58784*	-	-	-	-	-

**Table 3:** *trans-*Prenyltransferase genes and isoprenoid pathway gene co-expressions in *A. thaliana*.

**CYTOSOL AND MITOCHONDRIA**										
**Pathway**			**MVA**	**Sterol**	**Triterpenoids**	**Sesquiterpenoids/Diterpenoids**			**Protein Prenylation**	**Ubiquinone**
Total No. of Genes per Pathway			9	24	13	13			7	4
FPPS1		*At5g47770*	6	13	2	1			4	0
FPPS2		*At4g17190*	7	14	0	1			3	1
GGPPS1		*At1g49530*	0	0	2	0			0	3
GGPPS3		*At2g18640*	0	4	3	6			1	2
GGPPS11		*At4g36810*	0	11	2	0			1	0
GGPPS4		*At2g23800*	2	2	3	1			0	2
PPPS1		*At2g34630*	2	3	0	2			1	4
**PLASTIDS**										
**Pathway**		**MEP**	**Plastoquinone**	**Chlorophyll**	**Carotenoid**	**Phylloquinone**	**Gibberellins**	**Monoterpenoids**	**Diterpenoids/Sesquiterpenoids**	**Sesterterpenoids**
Total No. of Genes per Pathway		7	6	37	32	7	23	6	13	3
SSU	*At4g38460*	5	5	25	22	0	5	2	1	0
GGPPS11	*At4g36810*	7	3	29	18	1	7	1	0	0
GGPPS2	*At2g18620*	0	0	3	1	5	2	2	7	1
GFPPS1	*At3g14530*	0	0	1	0	3	2	0	5	2
GGPPS3	*At3g29430*	0	0	4	0	4	2	2	8	1
GGPPS4	*At3g32040*	0	0	3	0	5	7	2	7	1
PPPS2	*At3g20160*	0	0	4	0	4	4	2	8	1
PPPS1	*At2g34630*	0	0	0	2	4	2	4	2	1
SPPS1	*At1g78510*	6	6	29	20	0	4	2	1	0
SPPS2	*At1g17050*	7	4	29	22	0	7	2	1	0

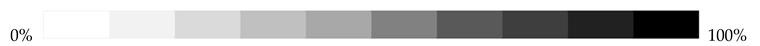
Table 3 shows number of pathway genes that are significantly co-expressed with the *trans-PTs*. The intensity of the highlighted background is directly proportional to the relative number of co-expressed genes/total number of pathway genes. Data for co-expression analysis were obtained from BAR/Expression Browser (http://bar.utoronto.ca/affydb/cgi-bin/affy_db_exprss_browser_in.cgi) and log2 transformed (see Table S2 for log2 transformed expression data). In general, AtGeneExpress_plus-Extended Tissue Series data/Average of replicate treatments were downloaded. Genes that are missing were not present on the microarrays, and therefore, they are not included in the analysis. Pearson correlation coefficients and the corresponding false discovery rate (FDR) *p*-values were calculated. The threshold for significance is *p*-value ≤ 0.05. A detailed description of the analysis can be found in [54]. Genes in categories mono-, di-, sesqui-, sester- and triterpenoids contain genes encoding only terpene cyclases. Genes in the category diterpenoids/sesquiterpenoids and sesquiterpenoids/diterpenoids partly overlap. In both categories were kept the genes that have no experimentally proven one or other activity, and plastidial genes were considered diterpenoids and kept only in the category diterpenoids/sesquiterpenoids.

## Supplementary Materials

The following are available online. Figure S1: Multiple sequence alignment for *A. thaliana cis*-PTs listed in Table 1; Table S1: Substrate and product specificity and kinetics of prenyltransferases in *A. thaliana*; Table S2: Gene expression data.
