# Supplementary material for: Functional Gene Network of Prenyltransferases in Arabidopsis thaliana
Source: Molecules. 2019 Dec 12;24(24):4556. doi: 10.3390/molecules24244556 (PMC6943727; doi:10.3390/molecules24244556)
Supplement: Supplementary file 1 [file molecules-24-04556-s001.zip › Figure S1.pdf]

```
CPT1_At2g23410    1 MLSL------------------------------------------------------------LSSDSSLL
CPT2_At2g23400    1 MLSL------------------------------------------------------------LST---LV
CPT6_At5g58780    1 MLSI------------------------------------------------------------LSS---LL
CPT7_At5g58770    1 MLSL-------------------------------------------RVPTPTSFDFRRYQAGDLERRWRLS
CPT8_At5g58782    1 MNFMIHANHLLVLFTLSTM---------------------------------------------LSMLWFLL
CPT9_At5g58784    1 MLSL------------------------------------------------------------FSVVFTFL
CPT3_At2g17570    1 MAEL-------------------------------------------PGQIRHIGGRMSQLLEQIYGFS---
CPT4_At5g60510    1 MNNT-------------------------------------------REEV----GEFTQIFNALMSLM---
CPT5_At5g60500    1 MN-T-------------------------------------------LEEV----DESTHIFNALMSLM---
LEW1_At1g11755    1 MDSNQSMRLLSAWIGQIGDLGLNLLWRFIHIVVSLWYIVSGIFEAIE-------------------------

CPT1_At2g23410   13 SLLFLFLIPCLFITSYIGFPVF--LLKLIGLIKIKAARD---NEKRDEGTYVVREDG-----LQRELMPR
CPT2_At2g23400   10 ALPFLFLIPCLFITSYICFPVF--LTKLLGLIKFKAARDDDDNEKRDEATCVVREEE-----LQRELMPR
CPT6_At5g58780   10 SLLFLFIISCFFITSHFWFPLS--LPKILGFIKITSSRDDYDNEQRDEGTYVVGVEE-----LQRELMPR
CPT7_At5g58770   30 RDSFLSFSP--------------------KFEENRGFRFGVKSSKSDVSFTAAEEEETLPEELHAELMPK
CPT8_At5g58782   28 SLLSLLLLPCL----RPCFPAKGSLK---------------NKKKIDKGTYVVGEEET-PKELQRELMPR
CPT9_At5g58784   13 AL---FLIPGLFISRRLNVPLS--LTNILRFIKIIASKYD-DEEERNEKRGTMGEKQK-RG---RNIMPK
CPT3_At2g17570   27 -------------------------------------------------------RRSLFRVISMGPIPC
CPT4_At5g60510   23 -------------------------------------------------------RKFIFKVLRVGPIPT
CPT5_At5g60500   22 -------------------------------------------------------RKFLFRVLCVGPIPT
LEW1_At1g11755   48 ----------------------------------------------------------------------

CPT1_At2g23410   73 HVAFILDGNRRWA-----KRAGL--TTSQGHEAGAKRLIDIAELCFELGVHTVSAFAFSTENWGRDKIEI
CPT2_At2g23400   73 HVSFILDGNRRWA-----KRDGL--TTAQGHEAGTKRIIEIAEVCFELGIHTVSAFAFSTENWGRDKFEV
CPT6_At5g58780   73 HVAVIMDGNRRWA-----KRAGL--LTSQGHEAGAKRLIEFSELCFKLGIHTVSAFAFSTENWGRHKIEV
CPT7_At5g58770   80 HVAIIMDGNGRWA-----KNRGL--QPWDGHRAGVEALKEIVELCGKWGIQVLTVFAFSTDNWIRPRIEI
CPT8_At5g58782   78 HVAVIMDGNRRWA-----KQTGL--LTSQGYEAGAKRLLEFADLCFKLGINTVSAFAFSTENWGRHKIEV
CPT9_At5g58784   73 HVAVILDGNRRWA-----EKRGL--GTSEGHEAGARRLMENAKDCFAMGTNTISLFAFSTENWERPEDEV
CPT3_At2g17570   42 HIAFIMDGNRRYA-----KKCGL--LDGSGHKAGFSALMSMLQYCYELGIKYVTIYAFSIDNFRRKPEEV
CPT4_At5g60510   38 NISFIMDGNRRFA-----KKRNLEGLD-AGHRAGFISVKYILQYCKEIGVPYVTLYAFGMDNFKRGPEEV
CPT5_At5g60500   37 NISFIMDGNRRFA-----KKHNLIGLD-AGHRAGFISVKYILQYCKEIGVPYVTLHAFGMDNFKRGPEEV
LEW1_At1g11755   48 SYAITLGLNKKYGSIDLEKLRCLAVVVDIEAAQDVANVVELLQWLTTIGVKQVGLF----DSQGLLKKSK
                       I                  II                   III

CPT1_At2g23410  136 DNLM-----SLIQHYRNKS-----N---IKFFHRSEVRVSVIGNKTKIPESLLKEIH-EIEEATKGYKNK
CPT2_At2g23400  136 KCLM-----SLFNHYL-KS-----N---IQYFQRKEVRVSVIGNKTKIPESLLKEIH-EIEEATKATR--
CPT6_At5g58780  136 KCLM-----SLIQHYL-KS-----K---IQYFQREETRVSVIGNLTKIPESLLRTVQ-EIEEATRSYKKK
CPT7_At5g58770  143 DFLF-----SLFERSL-KT-----E---FQNLAKNNVRISIIGDSSKLPKSLLRVIN-EVEEVTKNNTRL
CPT8_At5g58782  141 KCLM-----YLFQRYLKSK---------IQFFQSKEIRVSVIGNLAKIPESLLRTVH-ELEEATKSYKKK
CPT9_At5g58784  136 KCLM-----ALFEKYL-AS-----D---MPYLRSDKIKISVIGNRTKLPESLLGLIE-EVEEATKSYEGK
CPT3_At2g17570  105 ESVM-----DLMLEKIKSL---LEK---ESIVHQYGIRVYFIGNLALLNDQVRAAAE-KVMKATAKNSRV
CPT4_At5g60510  102 KCVM-----DLMLEKVELT---IDQ---AVSGNMNGVRIIFAGDLNSLNERFRAATK-KLMELTEENRDL
CPT5_At5g60500  101 KCVM-----DLMLEKVELA---IDQ---AVSGNMNGVRIIFAGDLDSLNEHFRAATK-KLMELTEENRDL
LEW1_At1g11755  114 DLILETVPGSMLLEEIEKDVAPDGKRIALEFISSSDNKEAVMKAANILLQRYLKSSHPEDDKG-------

CPT1_At2g23410  192 HLIMAVDYSGKFDIMHACK-SLVKKSEK------------------------GLIREEDVDEALIERELL
CPT2_At2g23400  189 ------------ISISSW-HLVKKSEK------------------------GLIREEDVDEALIERELL
CPT6_At5g58780  191 HLILAIDYSGRLDILRACK-SIVKKSEK------------------------GLIREEDVDEALIERELL
CPT7_At5g58770  198 QLIVAVGYSGKYDVLQACR-GIARRVKD------------------------GEIEVEEIDERLIEEELE
CPT8_At5g58782  196 HLILAIDYSGRFDILGAC-KNIVKKSEQ------------------------GLIREEDVDETLFERELQ
CPT9_At5g58784  191 NLIIAIDYSGRYDILQACK-SLANKVKD------------------------GLIQVEDINEKAMEKELL
CPT3_At2g17570  163 VLLICIAYNSTDEIVQAVKKSCINKSDNIEASNYKHEDSDSDIEGTDMENQEKKIQLVDIEENMQ-----
CPT4_At5g60510  160 IVVVCVAYSTSVEIVHAVRDSCVRKSKT----------------GDGSS----ALELSDIEECMY-----
CPT5_At5g60500  159 IVVVCVAYSTSLEIVHAVRKSCVRKCTN----------------GDDLV----LLELSDVEECMY-----
LEW1_At1g11755  177 -----------------------------------------------------------EDFFTESHLNDALR
                            IV

CPT1_At2g23410  237 TNCSDFPSPDLMIRTSGEQRISNFFLWQLAYSELFFSPVFWPDFDKDKLLEALASYQRRERRFGCRV---
CPT2_At2g23400  221 TNCSDFPSPDLMIRTSGEQRISNFFLWQLAYTELFYSPVLWPDFDKDKLLEALASYQGRERRFGCRV---
CPT6_At5g58780  236 TNCTEFPSPDLLIRTSGEQRISNFFLWQLAYTELFFSPVLWPDFDKDKLLEALVSYQRRERRFGCRV---
CPT7_At5g58770  243 TNCTEFPYPDLLIRTSGELRVSNFLLWQLAYTELFFAQELWPDFGRSGFIEALMSFQQRQRRFGGRKS--
CPT8_At5g58782  241 TRCTEFPSPDLLIRTSGEQRISNFFLWQLAYTEFFFSPVLWPDFDKQKFIEALVSYQRRDRRFGSRL---
CPT9_At5g58784  236 TKCSEFPNPDLLIRTSGEQRISNFFLWQSAYTELYFPTVLWPDFGEAEYLEALTWYQQRQRRFGRRV---
CPT3_At2g17570  228 --MSVAPNPDILIRSSGETRLSNFLLWQTGNTQLCSPAALWPEIGLRHLLWAILNFQRNHSYLEKRKKQL
CPT4_At5g60510  205 --TSVVPVPDLVVRTGGGDRLSNFMTWQTSRALLHRTEALWPELGLWHLVWAILKFQRMQDYLQKKKKLH
CPT5_At5g60500  204 --TSIVPVPDLVIRTGGGDRLSNFMTWQTSRSLLHRTEALWPELGLWHLVWAILKFQRMQDYLTKKKKLD
LEW1_At1g11755  191 VVGENVHVPDLLLVYGPIRSHLGFPAWRLRYTEIVHMGTL-KYMRYGSLLKAIHKFTGVHQNYGT-----
                                    V                                      RXG
                                                                           NXG
```

Figure S1. Multiple sequence alignment for *Arabidopsis cis*-PTs listed in Table 1. Highly conserved regions are higlighted in gray. They are not present in CPT binding protein LEW1. Motifs essential for binding of substrate are highlighted in black. RXG (eventually NXG) motif highlighted in green is critical for catalysis [65] and is missing in heteromeric CPT enzymes.
